# The Confinement Behavior and Mechanistic Insights of Organic Phase Change Material Encapsulated in Wood Morphology Genetic Nanostructures for Thermal Energy Storage

**DOI:** 10.3390/polym16223213

**Published:** 2024-11-20

**Authors:** Yang Meng, Yanping Jiang, Yuhui Chen, Jiangyu Zhang

**Affiliations:** Yunnan Provincial Key Laboratory of Energy Saving in Phosphorus Chemical Engineering and New Phosphorus Materials, The International Joint Laboratory for Sustainable Polymers of Yunnan Province, The Higher Educational Key Laboratory for Phosphorus Chemical Engineering of Yunnan Province, Faculty of Chemical Engineering, Kunming University of Science and Technology, Kunming 650500, China; yanpingjiang@stu.kust.edu.cn (Y.J.); yuhuichen@stu.kust.edu.cn (Y.C.); zhangjiangyu@stu.edu.cn (J.Z.)

**Keywords:** wood, morphology genetic nanostructures, phase change materials, thermal energy storage, nanoconfinement effect

## Abstract

Wood, a renewable and abundant biomass resource, holds substantial promise as an encapsulation matrix for thermal energy storage (TES) applications involving phase change materials (PCMs). However, practical implementations often reveal a disparity between observed and theoretical phase change enthalpy values of wood-derived composite PCMs (CPCMs). This study systematically explores the confinement behavior of organic PCMs encapsulated in a delignified balsa wood matrix with morphology genetic nanostructure, characterized by a specific surface area of 25.4 ± 1.1 m^2^/g and nanoscale pores averaging 2.2 nm. Detailed thermal performance evaluations uncover distinct phase change behaviors among various organic PCMs, influenced by the unique characteristics of functional groups and carbon chain lengths. The encapsulation mechanism is primarily dictated by host–guest interactions, which modulate PCM molecular mobility through hydrogen bonding and spatial constraints imposed by the hierarchical pore structure of the wood. Notably, results demonstrate a progressive enhancement of nanoconfinement effects, evidencing a transition from octadecane to stearic acid, further supported by density functional theory (DFT) calculations. This research significantly advances the understanding of nanoconfinement mechanisms in wood-derived matrices, paving the way for the development of high-performance, shape-stabilized composite PCMs that are essential for sustainable thermal energy storage solutions.

## 1. Introduction

Thermal energy constitutes a major part of global energy usage, accounting for more than half of all energy consumption [1]. In contemporary engineering applications such as the utilization of clean thermal sources like solar and geothermal energy [2,3], waste heat recovery and conversion [4], building energy efficiency [5], biomedical thermotherapy [6], thermal insulation fabrics [7], and thermal regulation of batteries [8], there is an escalating requirement for innovative energy storage solutions. Thermal energy storage (TES) systems, designed to store erratic and unpredictable energy as heat for subsequent use, are garnering significant interest for their potential to balance energy availability with demand [9]. This interest spans across sensible, latent, and thermochemical heat storage methods. Notably, as the core of the TES system is dominated by solid–liquid latent heat storage, organic materials including polyethylene glycol (PEG), paraffin, and fatty acids, are prized for their high energy storage capacity, minimal temperature variance, robust chemical stability, safety, and affordability, enabling them to effectively buffer intermittent energy flows [10]. However, the practical deployment of these phase change materials (PCMs) often encounters challenges such as morphological instability and susceptibility to liquid phase leakage, which always limit their effectiveness and applicability in real-world scenarios [11].

To prevent leakage in PCMs, integrating a secondary matrix and constructing form-stable composite PCMs (CPCMs) through techniques like microencapsulation [12], electrospinning [13], 3D printing [14], and porous media adsorption [15] have proven effective. Among these methods, the adsorption approach, which utilizes capillary forces, surface tension, or hydrogen bonding within micro- and nanoporous structures, is particularly favored for its simplicity, cost-effectiveness, and broad applicability. Significant research efforts have been dedicated to porous substrates like carbon foams/aerogels [16], synthetic foamable polymers (e.g., polyurethane and EVA) [17,18], 2D nanomaterials (e.g., graphene, MXene, and phosphorene) [19,20,21], and nanoclays [22] for the support of PCMs. Recently, there has been a growing interest in natural biomass resources like wood, bamboo, and rice husks [23,24,25]. This is because these materials could retain their natural pore structures via a top-down process, making them ideal for creating eco-friendly, three-dimensional composite PCMs with high encapsulation efficiency. Among biomass resources, wood is particularly abundant and versatile. The xylem of natural wood, with its network of capillaries (vessels and tracheids) and microcapillaries (pits), provides an excellent structure for encapsulating and stabilizing PCMs [26]. For instance, Liu et al. achieved encapsulation efficiencies of 83.9%, 84.0%, and 74.1% for myristic acid, paraffin, and polyethylene glycol, respectively, in chemically treated wood through a vacuum impregnation method [27]. Similarly, Pan et al. explored the use of carbonized wood as a support material, achieving an impregnation ratio of 85.25 wt% and demonstrating a high photo-thermal conversion efficiency of 90% [28]. In our previous study, balsa wood was used as the starting material to prepare the encapsulation-enhanced CPCMs via a combination of chemical modification and the integration of organic/inorganic interfacial modifiers such as boron nitride (BN), MXene, polypyrrole (PPy), and phytic acid (PA) [29,30]. However, we found that when organic PCMs are encapsulated in wood-based matrices, the actual phase change enthalpy often falls short of expectations. This discrepancy increases with chemical modifications, such as lignin removal, which always poses a challenge for using wood as an effective encapsulation matrix for PCMs.

As a protective porous medium, the pore structure, type, and surface chemical properties of the material play a crucial role in determining the thermal performance of encapsulated phase change materials (PCMs). Typically, smaller pores induce a “confinement effect”, which constrains the crystallization and orientation of PCM molecular chains [31]. Additionally, active functional groups on the surface of the porous medium can form intermolecular interactions, such as hydrogen bonding, with PCM molecules [32]. These interactions impede the mobility of PCM molecules, thereby reducing the number of molecules that actively participate in the phase change process. For instance, Kittaka et al. found that in porous media rich in surface hydroxyl groups (-OH), such as MCM-41 and SBA-15, when used as carriers to encapsulate polyethylene glycol (PEG), strong hydrogen bonds formed at the interface, restricting the movement of PEG molecules and almost completely suppressing the phase change behavior of the composite material [33]. In another study, Wang et al. modified the surface of SBA-15 to reduce hydrogen bonding at the interface by substituting the original -OH groups with NH_2_ and CH_3_ groups [34]. This modification preserved the adsorption capability while significantly reducing hydrogen bonding at the interface, resulting in PEG/NH_2_-SBA-15-CH_3_ composites with a higher phase change enthalpy. As for natural wood, by removing lignin and hemicellulose from lightweight balsa wood, the resulting wood-based framework, with its interconnected macroporous and microporous structure, significantly enhanced PEG encapsulation capacity. However, the removal of amorphous components while retaining the cellulose skeleton in wood-based encapsulation matrices may result in spatial confinement of the encapsulated phase change molecules due to the developed micro- and nanopore structure. Simultaneously, the abundant hydroxyl groups on the cellulose surface may form hydrogen bonds with the phase change molecules, leading to surface confinement effects. Nonetheless, our understanding of these confinement behaviors in wood-based materials remains speculative, with limited research on the topic, highlighting the need for in-depth exploration of the underlying mechanisms.

Herein, we present a delignified balsa-based matrix with a notable specific surface area of 25.4 ± 1.1 m^2^/g and a pore size of 2.2 nm, achieved through a two-step acidic sodium chlorite treatment [29]. This study systematically examines the thermal behavior of various organic phase change materials (PCMs) when confined within the nanoscale structure of cellulose nanofibrils. The results demonstrate distinct phase change behaviors among small molecular organic PCMs, each varying in functional groups and carbon chain lengths, which are primarily influenced by the nanoconfinement effects imposed by the hierarchical pores of the wood. The interactions at the host–guest interfaces within the composite PCMs play a crucial role in determining the extent of nanoconfinement. These interactions are significantly impacted by the mobility restrictions of PCM molecules, driven by the strength of hydrogen bonding and the spatial limitations of the pores. Our study reveals a progressive enhancement of nanoconfinement from octadecane to stearic acid, passing through octadecylamine and octadecanol. The atomic-level mechanisms behind these observations were further clarified through density functional theory (DFT) calculations. This research offers valuable insights into the nanoconfinement mechanisms of small molecular organic PCMs within wood-derived encapsulation matrix, paving the way for the development of high-performance, shape-stabilized composite PCMs for thermal energy storage applications.

## 2. Materials and Methods

### 2.1. Materials

Balsa wood was provided by Modulor New Materials GmbH, Berlin, Germany. Sodium chlorite, hydrogen peroxide, sodium hydroxide, glacial acetic acid, lauric acid (LA), myristic acid (MA), palmitic acid (PA), stearic acid (SA), octadecanol, octadecylamine, tetradecane, hexadecane, and octadecane were sourced from Sigma-Aldrich, Helsinki, Finland. Anhydrous ethanol and acetone were obtained from HyClone, Helsinki, Finland. Deionized water was prepared in the laboratory. All chemicals were of analytical grade, except for sodium chlorite (90% purity), sodium hydroxide, glacial acetic acid, anhydrous ethanol, and acetone, which were of analytical purity.

### 2.2. Preparation of Balsa Wood-Based Phase Change Composite Materials

Balsa wood was crushed into wood powder and sieved through a 40-mesh screen. After washing with deionized water and ethanol solution, the powder was dried in an oven at 103 ± 2 °C for later use. Five grams of the wood powder was added to 200 mL of acidic sodium chlorite solution (1% *w*/*w*, pH = 4.6) and stirred under mild boiling for 18 h. After the reaction, the delignified wood powder was washed multiple times until it turned pure white, and then dried for storage. The dried delignified wood powder was then added to a 1M sodium hydroxide solution, heated to mild boiling, and stirred vigorously for 6 h. The resulting product was washed, filtered, and freeze-dried to obtain the balsa wood powder encapsulation carrier material, designated as DBW. The modified balsa wood powder was mixed with organic phase change materials, including lauric acid (C12), myristic acid (C14), palmitic acid (C16), stearic acid (C18), tetradecane, hexadecane, octadecane, octadecanol, and octadecylamine, maintaining a mass fraction of 60%. This mixture was placed in a vacuum drying oven at 80 °C under −0.1 MPa to complete the encapsulation. After encapsulation, a leakage test was conducted by placing the composite phase change materials on filter paper in an 80 °C oven to remove any unabsorbed phase change materials, resulting in a stable balsa wood-based composite phase change material.

### 2.3. Methods

Detailed methods for the principal characterization techniques, and a comprehensive description of molecular dynamics simulations are provided in the Supplementary Materials Note S1.

## 3. Results and Discussion

### 3.1. Delignified Balsa-Based Encapsulation Matrix and Physicochemical Properties

**Figure 1:**
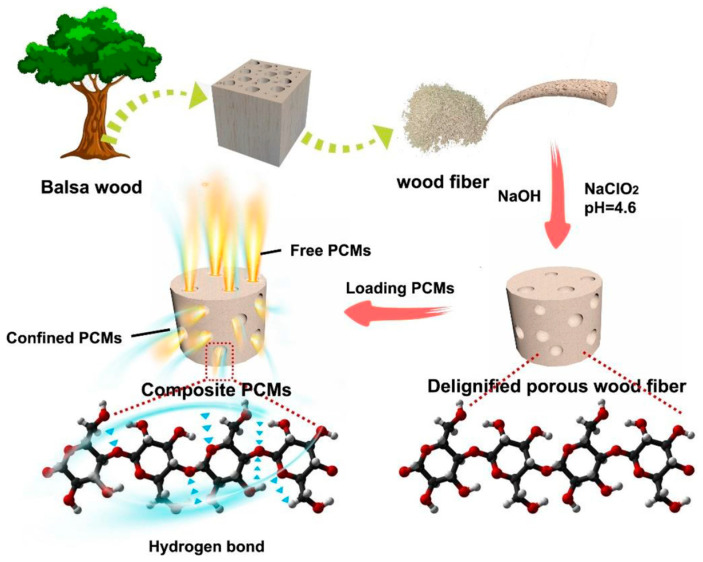
Fabrication and schematic of the balsa-based encapsulation matrix for thermal energy storage.

Figure 1 illustrates the delignification process of the balsa-based encapsulation matrix and the synthesis of the CPCMs. Typically, wood-derived 3D scaffolds, which preserve the natural large capillary channels, have been widely used as effective matrices for encapsulating organic PCMs [30]. However, the prominent large capillary structures within the wood can limit the confinement effect of PCMs within the smaller pore structures, such as mesopores and micropores. In these cases, the quantity of PCM encapsulated in the finer pores is significantly lower compared to the larger capillaries, which can lead to suboptimal performance. To overcome the drawbacks of these large pore structures (e.g., vessels and fiber tracheids) and enhance the confinement in smaller pores, the balsa wood was mechanically pulverized to disrupt its native large capillary channels. This process yielded a powder-like wood fiber comprising intertwined cellulose, hemicellulose, and lignin. Subsequent chemical treatments with acidic NaClO_2_ solution and NaOH solution successfully removed most of the lignin and nearly all the hemicellulose, leaving behind a stable, highly porous cellulose skeleton. This porous cellulose structure, enriched with micropores and mesopores, effectively encapsulates the PCMs through capillary forces, surface tension, and strong hydrogen bonding interactions formed by the abundant hydroxyl groups on its surface, thereby preventing leakage and enhancing encapsulation stability.

**Figure 2:**
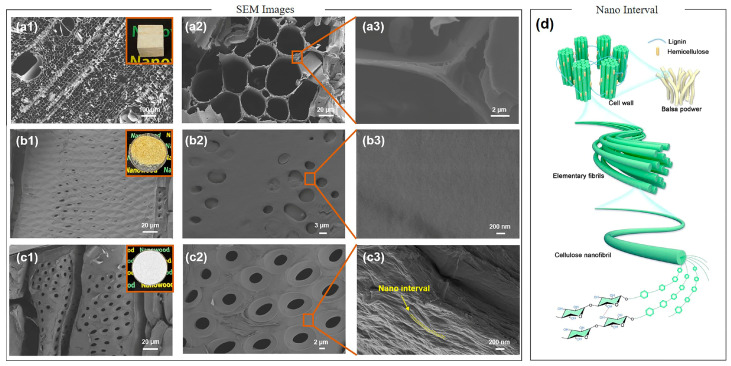
SEM images of (**a1**–**a3**) cross-sections of solid balsa wood, (**b1**–**b3**) balsa wood powder, and (**c1**–**c3**) delignified balsa wood powder; (**d**) a schematic representation of the multi-scale pore structure in wood.

The microstructure of solid balsa wood (RW) and balsa-derived powder (DBW) before and after delignified treatment is shown in Figure 2. It is evident that the RW exhibits a distinct capillary structure, including vessels approximately 150 μm in diameter and fiber tracheids measuring around 10–20 μm, which are typical features of hardwood (Figure 2(a1,a2)) [35]. Remarkably, these natural structures, especially the fiber tracheids with over 90% volumetric capacity and well-defined capillary dimensions, have been identified as promising encapsulation matrices for PCMs. However, despite the high encapsulation efficiency of wood-based matrices being over 80%, a mismatch between encapsulation efficiency and enthalpy has been reported, which is likely caused by the confinement effect of the cell walls (Figure 2(a3)) in contact with the PCM. As illustrated in Figure 2d, wood cell walls possess a complex, multi-scale structure, where cellulose nanofibers and hemicellulose are intertwined and coated by lignin, forming a networked framework [36]. We hypothesize that the micro- and nano-channels between oriented cellulose fibers, along with abundant surface hydroxyl groups, may influence the mobility of organic PCM molecules through spatial confinement and hydrogen bond anchoring, thereby affecting the phase transitions. To explore this hypothesis, we first subjected RW to mechanical pulverization, effectively disrupting its large capillary channels and exposing smaller-scale pore structures. As shown in Figure 2(b1,b2), the untreated RW powder displays a highly porous surface morphology, characteristic of its natural role in water and nutrient transport during growth. However, many pores are obstructed by thin membrane-like layers, and high-resolution imaging reveals irregular and partially sealed pore structures, impeding the full penetration of PCM into the wood powder. Following treatment with acidic NaClO₂ and subsequent alkali solutions, a significant portion of the lignin and nearly all hemicellulose were removed from the balsa wood powder [37]. This process completely disrupted the thin membrane covering the pore surfaces, fully exposing the pore structure, while causing cracks and defects in the overall fiber framework, leaving a highly porous structure (Figure 2(c1)). The pore size distribution was also altered, as shown in Figure 2(c2), where the average pore diameter expanded from 1–1.5 μm in the untreated wood to 1.5–2.0 μm after modification. Moreover, as seen in Figure 2(b3,c3), the surface morphology of the wood cell walls transitioned from smooth and compact to a wrinkled state, with distinct microfibril alignments, and small voids formed between the fibrils. These structural changes collectively enhanced the permeability of the modified balsa wood powder, creating a highly porous, multi-scale framework with increased specific surface area. These improvements significantly boosted the PCM encapsulation capacity, laying the groundwork for further research into the effects of micro- and nano-scale spatial confinement in wood-based matrices on PCM molecular dynamics.

**Figure 3:**
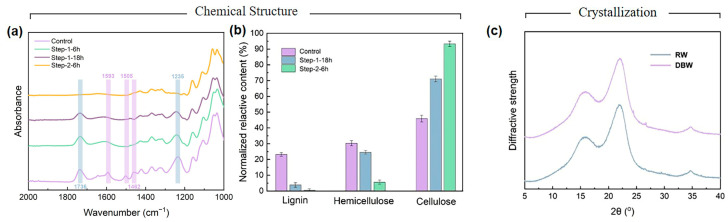
(**a**) FTIR spectra of modified balsa wood powder; (**b**) normalized composition (lignin, hemicellulose, and cellulose) for modified during different treatment process; (**c**) XRD Spectra of untreated and delignified balsa wood powder.

The FTIR spectra of RW at different stages of delignification are shown in Figure 3a. For untreated RW, two prominent absorption peaks are observed at around 1736 cm^−1^ and 1235 cm^−1^, corresponding to the stretching vibrations of C=O (carbonyl group) and CO-OR (ester group), which are characteristic peaks of the amorphous polysaccharides (hemicelluloses) in lignocellulosic materials [38]. Additionally, three weaker peaks appear at 1590 cm^−1^, 1505 cm^−1^, and 1462 cm^−1^, associated with the aromatic skeletal vibrations of lignin [39]. During the initial treatment with acidic sodium chlorite, a gradual decrease in the intensity of these lignin-related peaks is observed, reflecting the progressive removal of lignin. After approximately 18 h of treatment, the peaks at 1590 cm^−1^, 1505 cm^−1^, and 1462 cm^−1^ nearly vanish, indicating that most of the lignin has been successfully extracted. However, the intensity of the hemicellulose-related peaks at 1736 cm^−1^ and 1235 cm^−1^ only shows a slight reduction compared to the initial spectrum, demonstrating that the acidic sodium chlorite treatment selectively removes lignin while retaining most of the hemicellulose, consistent with the findings of Liu et al. [27]. In the second stage of treatment, after 6 h of exposure to an alkaline solution, the peaks at 1736 cm^−1^ and 1235 cm^−1^ disappear completely, indicating the almost complete removal of hemicellulose, leaving behind the cellulose framework of the cell walls. Subsequently, the content of cellulose, hemicellulose, and lignin at various reaction stages was quantified, as shown in Figure 3b. Untreated balsa wood contains approximately 23.6% lignin, 30.2% hemicellulose, and 46.2% cellulose. After 18 h of acidic sodium chlorite treatment, the relative lignin content decreases significantly to 5%, while hemicellulose content only slightly decreases to 27.7%. Following the alkaline treatment, the lignin content further drops to 1%, and the hemicellulose content decreases to 7%, leaving a high cellulose content of 92%. These results indicate that the two-step modification process, involving acidic sodium chlorite and alkaline solution treatments, effectively removes the majority of lignin and hemicellulose, leaving a cellulose-dominant structure.

X-ray diffraction (XRD) analysis was conducted to evaluate the impact of the treatment process on the cellulose structure of RW. As shown in Figure 3c, the untreated balsa wood powder displayed characteristic diffraction peaks at 2θ values of 16.0°, 22.0°, and 34.8°, corresponding to the (101), (020), and (040) crystallographic planes of cellulose, which are indicative of the cellulose I structure [40]. After treatment with acidic sodium chlorite and an alkaline solution, these characteristic peaks remained in the DBW, with peak positions and intensities similar to those of the untreated sample. Crystallinity was calculated using the Segal method, revealing that the crystallinity index remained largely unchanged following the modification process [41]. These findings demonstrate that the treatment effectively removed lignin and hemicellulose while maintaining the original crystalline structure of the cellulose framework. The abundant cellulose content, with its crystallinity preserved, reorganized and bonded through hydrogen interactions, forming a stable three-dimensional porous cellulose matrix, which is advantageous for the encapsulation of organic PCMs.

**Figure 4:**
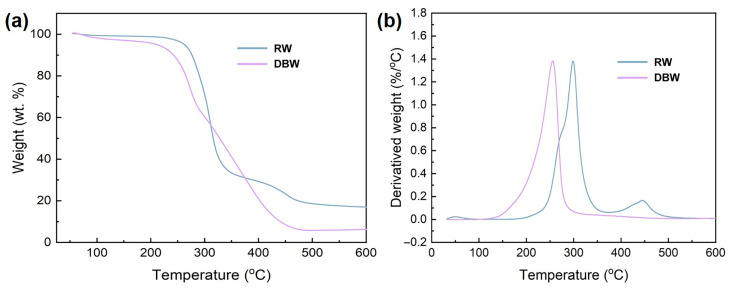
(**a**) TGA and (**b**) DTG of balsa wood powder before and after delignification.

The thermal decomposition behavior of untreated and lignin/hemicellulose-free balsa wood powder is illustrated in Figure 4. The TGA and DTG curves in Figure 4 reveal that the thermal degradation of RW occurs in three distinct stages. It is evident that for RW, the first stage of thermal degradation is primarily associated with the decomposition of hemicellulose and a portion of the cellulose. Complete degradation of hemicellulose occurs at approximately 275 °C. The second stage, between 275 °C and 300 °C, corresponds to the decomposition of the main cellulose fraction. The third stage, beyond 300 °C, is predominantly the degradation of lignin. The presence of lignin contributes to the formation of char and ash, accounting for approximately 20% of the final mass, which is consistent with the typical pyrolysis behavior of lignocellulosic materials [42]. In contrast, the thermal degradation of DBW, from which lignin and hemicellulose have been removed, occurs in a single phase, dominated by the pyrolysis of cellulose. The absence of thermally stable lignin results in a faster degradation process, with the peak degradation rate of cellulose occurring at 250 °C, which is 25 °C lower than that of untreated balsa wood, indicating that the modified material is more thermally labile [43]. Furthermore, the residual char and ash content for the DBW is less than 5%, which can be attributed to the removal of lignin, a carbon-rich component that typically enhances char formation during pyrolysis. These results demonstrate that the treatment with acidic sodium chlorite, followed by alkaline solution, effectively removes lignin and hemicellulose, leaving behind a preserved cellulose framework.

**Figure 5:**
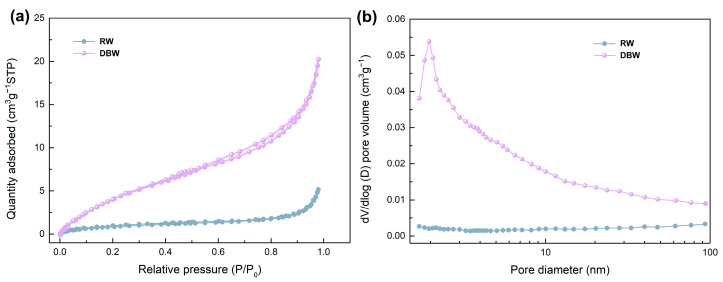
(**a**) Nitrogen adsorption–desorption isotherms and (**b**) pore size distribution of balsa wood before and after delignification.

The pore structure and distribution in the balsa wood powders are critical factors that influence the PCMs encapsulation performance. It is reported that a reduction in pore size enhances capillary forces, while an increase in pore volume helps improve the energy storage density of the encapsulated PCMs [15]. SEM images in Figure 2 already demonstrate that the two-step delignification process significantly enlarged the macroporous channels of the RW. To gain further insights into the pore structure across a range of scales, from micropores to macropores, both untreated and modified balsa wood powders were analyzed using BET surface area and pore size distribution methods. The N_2_ adsorption–desorption isotherms and pore volume distribution curves are shown in Figure 5. As illustrated in Figure 5a, both RW and DBW exhibit Type IV isotherms with a clear hysteresis loop, indicative of a porous structure containing micropores (<2 nm), mesopores (2–10 nm), and macropores (10–50 nm) [44]. After the removal of the amorphous component (lignin and hemicellulose), the N_2_ adsorption capacity of the modified balsa wood powder significantly increases compared to the untreated one. The gradual rise in N_2_ adsorption at P/P_0_ < 0.1 in the modified sample suggests the presence of micropores, while the sharp increase at P/P_0_ > 0.9 indicates the formation of macropores [45]. Figure 5b highlights that RW displays minimal micropore and mesopore distribution, with a modest increase in macropore volume. Conversely, the DBW exhibits a pronounced distribution of micropores and mesopores, indicating the formation of an extensive and well-developed porous network. This structural transformation results in a dramatic increase in specific surface area, from 3.2 ± 0.6 m^2^/g in the RW to 25.4 ± 1.1 m^2^/g in the DBW, while the average pore diameter decreases from 60.0 nm to 2.2 nm (Table 1). The substantial increase in surface area, coupled with the reduction in pore size, confirms the development of a sophisticated microporous, mesoporous, and macroporous architecture that is highly conducive to the efficient encapsulation of organic PCMs.

### 3.2. Evolution of the Chemical Structure in Balsa Powder-Based CPCMs

**Figure 6:**
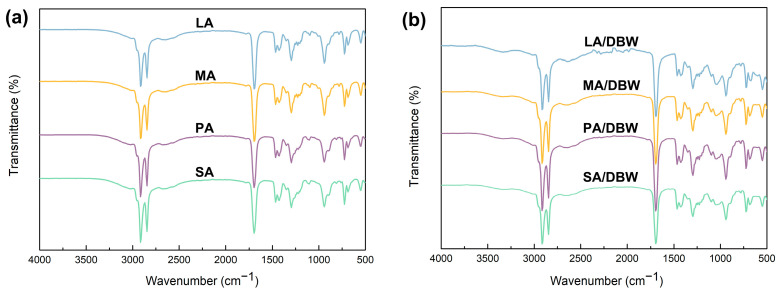
FTIR spectra of (**a**) fatty acids and (**b**) their CPCMs.

For the preparation of balsa powder-based CPCMs, a physical impregnation method was employed using fatty acid-derived PCMs with different molecular weights but similar functional groups. This study focused on the confinement effect of the modified balsa powder, post-delignification, due to changes in pore size during the encapsulation of the PCMs. Additionally, PCMs with identical carbon chain lengths but different terminal functional groups were selected to evaluate the effect of surface functional group modifications on the phase change properties of the encapsulated CPCMs after delignification. The FTIR spectra of both the pure PCMs and the corresponding composites were analyzed before and after encapsulation. Figure 6 displays the FTIR spectra of fatty acids with varying carbon chain lengths, while PCMs with the same carbon chain length but different functional groups, and their respective composites are shown in Figure S1. It can be observed that, compared to the pure PCMs, the characteristic peaks of the CPCMs in the DBW powder remain unchanged, with no new chemical bonds forming, and the peak positions remain consistent. This indicates that the interaction between the PCMs and DBW matrix occurs through physical adsorption, including hydrogen bonding, which restricts PCM molecular mobility without forming covalent bonds [46]. This form of physical adsorption effectively prevents leakage during phase transitions while preserving the original phase change properties of the materials.

**Figure 7:**
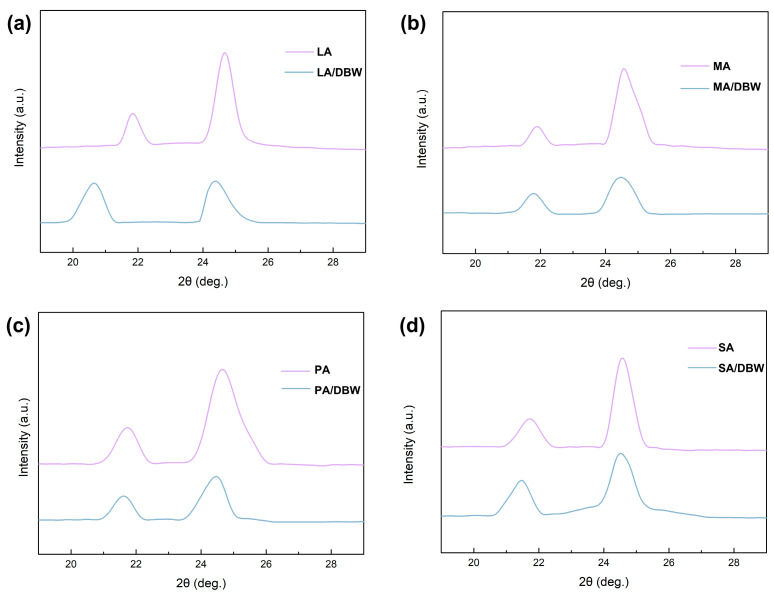
XRD curves of fatty acid and as-prepared composite phase materials: (**a**) LA, (**b**) MA, (**c**) PA, and (**d**) SA.

X-ray diffraction (XRD) analysis was conducted to evaluate the effect of the porous encapsulation matrix formed by DBW on the crystallization behavior of various PCMs. As shown in Figure 7, the characteristic peaks of the encapsulated PCMs exhibit varying degrees of low-angle shifts compared to their unencapsulated forms. This shift is likely due to the removal of lignin and hemicellulose, which exposes numerous hydroxyl (-OH) groups on the surface of the DBW. These groups can form hydrogen bonds with the PCM molecules at the interface, disrupting their orderly molecular arrangement and causing an expansion of the crystalline unit cells. According to Bragg’s law, the expansion of the unit cells leads to an increase in the intermolecular spacing, which results in a decrease in the diffraction angle (2θ), causing the characteristic peaks to shift toward lower angles in the XRD patterns [40]. The extent of this angular shift varies depending on the type of PCM, indicating that the strength of the interfacial interactions between the DBW and different PCMs differs. Additionally, Figure 7 shows a noticeable reduction in the relative diffraction intensity for the encapsulated PCMs compared to the unencapsulated ones. The reduction in relative diffraction intensity for the CPCMs suggests restricted crystallization, attributed to hydrogen bonding at the interface between DBW and PCM molecules. This type of hydrogen bonding interaction is considered physical adsorption here, as it limits molecular motion while maintaining the PCM’s chemical structure [32]. From the changes in the XRD patterns in Figure 7, it is evident that when the functional groups of the PCM molecules remain constant, but the molecular length varies, the confined space within the DBW limits the mobility of the PCM molecules, thus affecting their crystallization behavior. Similarly, as shown in Figure S2, when the molecular length of the PCMs is nearly identical, but the functional groups differ, the interfacial confinement effect between the DBW and the PCMs still influences the crystallization behavior. Despite the confinement effect caused by these two factors, the remaining free space within the DBW still allows for partial crystallization of the PCM. Therefore, the PCM molecules encapsulated within the DBW can still exhibit a certain degree of crystallinity.

### 3.3. Thermal Performance and Confinement Mechanism of Balsa Powder-Based CPCMs

To further investigate the confinement behavior of PCM molecules within the microchannels of DBW, the relative crystallinity of various CPCMs was calculated using Equation (1).

(1) $$F_{C}=\frac{\Delta H_{PCM}}{\Delta H_{pure}\beta }\times 100\%,$$

In this equation, Δ*H_Pure_* and Δ*H_PCM_* represent the latent heat of fusion for the pure PCM and the CPCMs, respectively (J/g), while *β* denotes the mass fraction of PCM encapsulated within the DBW. The confinement factor (*F_C_*) reflects the interaction between the PCM molecules and the wood-based encapsulation matrix.

Additionally, to eliminate the influence of varying encapsulation mass fractions on the experimental outcomes, all CPCMs were standardized to a 60% mass fraction based on the maximum encapsulation capacity of the delignified balsa wood powder. Table S1 summarizes the thermophysical properties of various pure PCMs. It is evident that as the carbon chain length of the fatty acids increases, both the melting and freezing points rise, and the corresponding melting and crystallization enthalpies increase accordingly. The thermal properties of the wood-based CPCMs are detailed in Table S2. As hypothesized, the actual phase change behavior (enthalpy values) of the CPCMs was lower than the theoretically calculated values, indicating that the motion of the PCM molecules was indeed restricted within the microchannels of the DBW. The calculated relative crystallinity values were all less than 100%, further confirming that the balsa wood encapsulation matrix imposes a confinement effect, limiting the movement of the organic PCM molecules.

**Figure 8:**
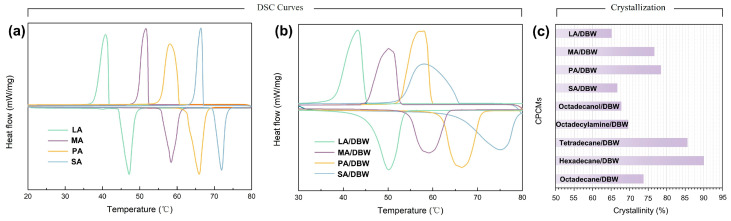
DSC of (**a**) fatty acid and (**b**) as-prepared composite phase change material; (**c**) crystallinities of composite PCMs.

The DSC curves of PCMs concerning fatty acid with the same terminal functional group (carboxyl) but varying carbon chain lengths, including LA, MA, PA, and SA, as well as the as-prepared wood-based CPCMs, are shown in Figure 8a,b, respectively. The DSC profiles of the wood-based CPCMs closely resemble those of the pure fatty acids, indicating that the phase change behavior of the PCMs is largely preserved within the confined structure of the DBW. As shown in Figure 8c and Table S2, the actual latent heat values (enthalpy of fusion and crystallization) for the encapsulated PCMs increase as the carbon chain length extends from C12 to C16. However, this trend reverses for stearic acid (C18), where the actual phase change enthalpy falls below that of palmitic acid (C16). According to Tables S1 and S2, pure SA exhibits higher melting and crystallization enthalpy compared to PA, but when encapsulated within the DBW matrix at 60% mass fraction, the phase change enthalpy of SA is lower. This trend is further reflected in the relative crystallinity values, where the C16 composite exhibits a crystallinity of 78.44%, while the C18 composite shows a lower crystallinity of 66.65%. The decrease in phase change enthalpy with increasing carbon chain length in the encapsulated composites can be attributed to two main confinement mechanisms: interfacial hydrogen bonding and the reduced average pore size. In the DBW matrix, strong hydrogen bonds at the interface ensure the tight adhesion of PCM molecules to the pore surfaces, enhancing encapsulation efficiency. However, these interactions also restrict the free thermal motion of the PCM molecules during phase transitions, leading to a reduction in enthalpy and crystallinity. Furthermore, the reduction in pore size within the modified balsa wood matrix amplifies capillary forces, which enhance the retention of the PCMs within the pores. However, when the average pore size approaches the molecular length of the PCM molecules, the molecules become confined within the pores, restricting their thermal mobility during phase transitions. This confinement effect further reduces the actual enthalpy and crystallinity. The balance between these two factors (interfacial hydrogen bonding and molecular confinement within the pores) varies depending on the carbon chain length of the fatty acids.

As shown in Table S2, the crystallization behavior of organic fatty acids encapsulated in the DBW reveals an increasing trend in relative crystallinity as the carbon chain length extends from C12 to C16, with values rising from 65.14% to 78.44%. This increase is primarily attributed to the dominance of hydrogen bonding interactions. Since the loading rate for all fatty acids in this study was fixed at 60%, shorter carbon chains imply a higher number of fatty acid molecules, which in turn increases the number of available adsorption sites. The removal of lignin and hemicellulose exposes a substantial number of hydroxyl (-OH) groups on the balsa wood surface, which act as binding sites, reinforcing hydrogen bonding at the interface. The increase in molecular count strengthens these hydrogen bonds, restricting the free thermal motion of the fatty acid molecules and thereby reducing the relative crystallinity of the encapsulated composite materials. However, when the carbon chain length extends from C16 to C18, the relative crystallinity of the encapsulated composite decreases, falling from 78.67% to 66.65%. This decline can be attributed to spatial confinement effects resulting from the reduced pore size. As mentioned earlier, when the average pore size of the encapsulation matrix closely matches the molecular size of the PCM, the organic fatty acid molecules become confined within the pore structure, limiting their thermal mobility. This confinement reduces both the phase change performance and crystallinity of the encapsulated materials. For SA (C18), which has an average molecular length of 2.2 nm (Table S3), the size closely matches the 2.2 nm average pore diameter of the DBW. Consequently, strong spatial confinement occurs, significantly restricting the thermal motion of SA molecules, resulting in a noticeable reduction in relative crystallinity. To further validate this confinement effect, phase change behavior was tested using tetradecane, hexadecane, and octadecane encapsulated in the composite materials, which have different functional groups. Relative crystallinity was calculated from the DSC results, and as shown in Figure 8c, when the carbon chain length increased from C14 to C16, the relative crystallinity rose from 85.58% to 89.98%. However, as the carbon chain length increased from C16 to C18, the relative crystallinity decreased from 89.98% to 73.77%. This behavior closely mirrors the results seen in the fatty acid-based PCMs, reinforcing the validity of the spatial confinement mechanism.

**Figure 9:**
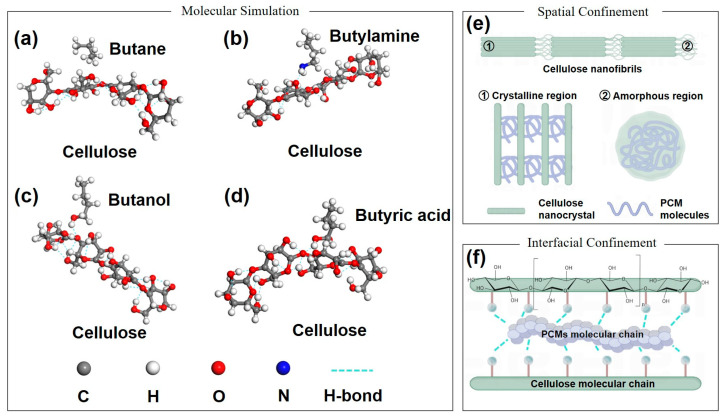
Double molecular models of phase change materials and cellulose: (**a**) butane, (**b**) butylamine, (**c**) butanol, and (**d**) butyric acid; schematic diagram of (**e**) spatial and (**f**) interfacial confinement mechanism.

Beyond elucidating the spatial confinement mechanism within the modified balsa wood matrix, further investigation focused on the interfacial confinement mechanism. This study examined the effect of hydrogen bonding at the interface between organic PCMs and the DBW on the thermal motion of PCM molecules. PCMs with identical carbon chain lengths but different functional groups including octadecane, octadecylamine, octadecanol, and stearic acid were selected for this investigation. Since these molecules share the same number of carbon atoms, they possess similar molecular sizes, effectively eliminating spatial confinement effects. Differences in their confinement behavior thus arise from variations in interfacial hydrogen bonding strength. To gain deeper insights into these interfacial interactions, density functional theory (DFT) was employed to calculate the binding energy (*E_int_*) between PCM molecules and cellulose. Due to the negligible impact of carbon chain length on the binding energy between octadecane, octadecylamine, octadecanol, stearic acid, and cellulose, shorter analogs such as butane, butylamine, butanol, and butyric acid were used for simplified calculations. Simulations performed using Material Studio software (2020), involved geometry optimization under the Compass II force field, with molecular models presented in Figure 9. Results in Figure 9a–d reveal that butane formed minimal hydrogen bonds with cellulose, while butylamine, butanol, and butyric acid showed substantial hydrogen bonding interactions. Binding energies, summarized in Table S4, quantify the strength of these interactions. Lower binding energy indicates greater system stability and stronger molecular interaction [47]. The order of binding strength was as follows: butyric acid < butanol < butylamine < butane, signifying that stearic acid forms the strongest hydrogen bonds with the modified balsa wood matrix, followed by octadecanol, octadecylamine, and octadecane. Stronger hydrogen bonding leads to enhanced confinement, restricting the thermal mobility of the PCM molecules and reducing phase change performance and crystallinity. This trend is evident in Table S2, where relative crystallinity declines from octadecane (73.77%) to octadecylamine (69.65%), octadecanol (67.77%), and stearic acid (66.65%). These findings validate the surface interfacial confinement mechanism (Figure 9e,f).

## 4. Conclusions

This study presents a comprehensive investigation into the confined phase change behavior of small organic PCMs within microporous and mesoporous wood-based encapsulation matrices. By using acidic sodium chlorite followed by alkaline treatment, the delignified balsa wood matrix develops a high specific surface area and a well-distributed micro- and nanoporous structure. These structural features enhance the encapsulation capacity and also have an impact on the thermal performance of PCMs. Encapsulation of PCMs with identical carboxyl groups but varying carbon chain lengths, as well as PCMs with the same carbon chain length but different functional groups, revealed strong interactions between the PCMs and the modified balsa wood matrix, as evidenced by peak shifts in DSC analysis. Combined with DFT simulations, the results identified two key mechanisms of confinement: spatial confinement, driven by the alignment between pore size and molecular dimensions, and surface confinement, governed by hydrogen bonding at the interface between PCM molecules and the matrix. These interactions limit the thermal mobility of the PCMs, leading to reduced phase change capacity and crystallinity. The findings underscore that optimizing the surface chemistry and pore architecture of the wood-based matrix is critical for enhancing encapsulation efficiency and minimizing confinement, ultimately improving the thermal performance of PCM composites for advanced energy storage applications.

## Figures and Tables

**Table 1:** Specific surface area and average pore size of balsa powder before and after delignification.

Samples	Density (kg/m^3^)	Specific Surface Area (m^2^/g)	Mean Pore Diameter (nm)
RW	90.5 ± 2.7	3.2 ± 0.6	60.0
DBW	29.7 ± 1.6	25.4 ± 1.1	2.2

## Data Availability

Data are contained within this article.

## Supplementary Materials

The following supporting information can be downloaded at: https://www.mdpi.com/article/10.3390/polym16223213/s1, Note S1: The Detailed Experimental Methods; Figure S1: FTIR spectra of (a) phase change material with different functional groups and (b) the as-prepared composite; Figure S2: XRD curves of fatty acid and as-prepared composite phase materials: (a) octadecane, (b) octadecylamine, (c) octadecanol, and (d) stearic acid (SA). Figure S3: DSC of (a) phase change material with different functional groups and (b) the as-prepared composite; Table S1: The thermal physical properties of pure organic phase change materials; Table S2: The thermal physical properties of wood-based CPCMs; Table S3: PCMs molecular sizes; Table S4: The bonding energy of PCMs and cellulose, ref. [48] is contained in supplementary materials.

## References

[1] Sulaiman N.S., Mohamad Amini M.H. (2022). Review on the Phase Change Materials in Wood for Thermal Regulative Wood-Based Products. Forests.

[2] Gupta M., Ashy (2024). Solar Thermal Energy Storage Systems Based on Discotic Nematic Liquid Crystals That Can Efficiently Charge and Discharge below 0 °C. Adv. Energy Mater..

[3] Shah M., Prajapati M., Yadav K., Sircar A. (2024). A comprehensive review of geothermal energy storage: Methods and applications. J. Energy Storage.

[4] Du K.-W., Wu C.-I. (2024). An Innovative Tubular Thermoelectric Generator (TTEG) for Enhanced Waste Heat Recovery in Industrial and Automotive Applications. Appl. Sci..

[5] Can A., Žigon J. (2022). n-Heptadecane-Impregnated Wood as a Potential Material for Energy-Saving Buildings. Forests.

[6] Chen J., Shi Y., Ying B., Hu Y., Gao Y., Luo S., Liu X. (2024). Kirigami-enabled stretchable laser-induced graphene heaters for wearable thermotherapy. Mater. Horiz..

[7] Asghari M., Fereidoni S., Fereidooni L., Nabisi M., Kasaeian A. (2024). Energy efficiency analysis of applying phase change materials and thermal insulation layers in a building. Energy Build..

[8] Liu K., Peng Q., Liu Z., Li W., Cui N., Zhang C. (2024). Adaptive battery thermal management systems in unsteady thermal application contexts. J. Energy Chem..

[9] Ali H.M., Rehman T.-u., Arıcı M., Said Z., Duraković B., Mohammed H.I., Kumar R., Rathod M.K., Buyukdagli O., Teggar M. (2024). Advances in thermal energy storage: Fundamentals and applications. Prog. Energy Combust. Sci..

[10] Umair M.M., Zhang Y., Iqbal K., Zhang S., Tang B. (2019). Novel strategies and supporting materials applied to shape-stabilize organic phase change materials for thermal energy storage—A review. Appl. Energy.

[11] Kahwaji S., White M.A. (2021). Organic Phase Change Materials for Thermal Energy Storage: Influence of Molecular Structure on Properties. Molecules.

[12] Alva G., Lin Y., Liu L., Fang G. (2017). Synthesis, characterization and applications of microencapsulated phase change materials in thermal energy storage: A review. Energy Build..

[13] Suárez-García A., Arce E., Alford L., Luhrs C.C. (2023). Electrospun composite fibers containing organic phase change materials for thermo-regulation: Trends. Renew. Sustain. Energy Rev..

[14] Yang J., Zhou Y., Yang L., Feng C., Bai L., Yang M., Yang W. (2022). Exploring Next-Generation Functional Organic Phase Change Composites. Adv. Funct. Mater..

[15] Liu Y., Zheng J., Deng Y., Wu F., Wang H. (2021). Effect of functional modification of porous medium on phase change behavior and heat storage characteristics of form-stable composite phase change materials: A critical review. J. Energy Storage.

[16] Maleki M., Imani A., Ahmadi R., Emrooz H.B.M., Beitollahi A. (2020). Low-cost carbon foam as a practical support for organic phase change materials in thermal management. Appl. Energy.

[17] Xi P., Xia L., Fei P., Zhang D., Cheng B. (2012). Preparation and performance of a novel thermoplastics polyurethane solid–solid phase change materials for energy storage. Sol. Energy Mater. Sol. Cells.

[18] Zhang M., Wang C., Luo A., Liu Z., Zhang X. (2020). Molecular dynamics simulation on thermophysics of paraffin/EVA/graphene nanocomposites as phase change materials. Appl. Therm. Eng..

[19] Kalidasan B., Pandey A.K., Rahman S., Yadav A., Samykano M., Tyagi V.V. (2022). Graphene–Silver Hybrid Nanoparticle based Organic Phase Change Materials for Enhanced Thermal Energy Storage. Sustainability.

[20] Solangi N.H., Mubarak N.M., Karri R.R., Mazari S.A., Jatoi A.S., Koduru J.R., Dehghani M.H. (2022). MXene-based phase change materials for solar thermal energy storage. Energy Conv. Manag..

[21] Aftab W., Khurram M., Jinming S., Tabassum H., Liang Z., Usman A., Guo W., Huang X., Wu W., Yao R. (2020). Highly efficient solar-thermal storage coating based on phosphorene encapsulated phase change materials. Energy Storage Mater..

[22] Voronin D.V., Ivanov E., Gushchin P., Fakhrullin R., Vinokurov V. (2020). Clay Composites for Thermal Energy Storage: A Review. Molecules.

[23] Can A., Lee S.H., Antov P., Abd Ghani M.A. (2023). Phase-Change-Material-Impregnated Wood for Potential Energy-Saving Building Materials. Forests.

[24] Yue X., Zhang R., Jin X., Zhang X., Bao G., Qin D. (2023). Bamboo-derived phase change material with hierarchical structure for thermal energy storage of building. J. Energy Storage.

[25] Li C., Sun Z., Wang Y., Zhu J., Wu J., Feng L., Wen X., Cai W., Yu H., Wang M. (2024). A novel biogenic porous core/shell-based shape-stabilized phase change material for building energy saving. J. Energy Storage.

[26] Jiang T., Zhang Y., Olayiwola S., Lau C., Fan M., Ng K., Tan G. (2022). Biomass-derived porous carbons support in phase change materials for building energy efficiency: A review. Mater. Today Energy.

[27] Liu S., Wu H., Du Y., Lu X., Qu J. (2021). Shape-stable composite phase change materials encapsulated by bio-based balsa wood for thermal energy storage. Sol. Energy Mater. Sol. Cells.

[28] Pan X., Zhang N., Yuan Y., Shao X., Zhong W., Yang L. (2021). Balsa-based porous carbon composite phase change material with photo-thermal conversion performance for thermal energy storage. Sol. Energy.

[29] Shi X., Meng Y., Bi R., Wan Z., Zhu Y., Rojas O.J. (2022). Enabling unidirectional thermal conduction of wood-supported phase change material for photo-to-thermal energy conversion and heat regulation. Compos. B Eng..

[30] Chen Y., Meng Y., Zhang J., Xie Y., Guo H., He M., Shi X., Mei Y., Sheng X., Xie D. (2024). Leakage Proof, Flame-Retardant, and Electromagnetic Shield Wood Morphology Genetic Composite Phase Change Materials for Solar Thermal Energy Harvesting. Nanomicro Lett..

[31] Tong X., Yang P., Zeng M., Wang Q. (2020). Confinement Effect of Graphene Interface on Phase Transition of n-Eicosane: Molecular Dynamics Simulations. Langmuir.

[32] Qian T., Li J., Min X., Fan B. (2018). Integration of Pore Confinement and Hydrogen-Bond Influence on the Crystallization Behavior of C18 PCMs in Mesoporous Silica for Form-Stable Phase Change Materials. ACS Sustain. Chem. Eng..

[33] Kittaka S., Ishimaru S., Kuranishi M., Matsuda T., Yamaguchi T. (2006). Enthalpy and interfacial free energy changes of water capillary condensed in mesoporous silica, MCM-41 and SBA-15. Phys. Chem. Chem. Phys..

[34] Wang J., Yang M., Lu Y., Jin Z., Tan L., Gao H., Fan S., Dong W., Wang G. (2016). Surface functionalization engineering driven crystallization behavior of polyethylene glycol confined in mesoporous silica for shape-stabilized phase change materials. Nano Energy.

[35] Qi Y., Jiang B., Lei W., Zhang Y., Yu W. (2024). Reliability Analysis of Normal, Lognormal, and Weibull Distributions on Mechanical Behavior of Wood Scrimber. Forests.

[36] Song J., Chen C., Zhu S., Zhu M., Dai J., Ray U., Li Y., Kuang Y., Li Y., Quispe N. (2018). Processing bulk natural wood into a high-performance structural material. Nature.

[37] Li K., Wang S., Chen H., Yang X., Berglund L.A., Zhou Q. (2020). Self-Densification of Highly Mesoporous Wood Structure into a Strong and Transparent Film. Adv. Mater..

[38] Fodor F., Hofmann T. (2024). Chemical Composition and FTIR Analysis of Acetylated Turkey Oak and Pannonia Poplar Wood. Forests.

[39] Bradai H., Koubaa A., Zhang J., Demarquette N.R. (2024). Effect of Wood Species on Lignin-Retaining High-Transmittance Transparent Wood Biocomposites. Polymers.

[40] Alqrinawi H., Ahmed B., Wu Q., Lin H., Kameshwar S., Shayan M. (2024). Effect of partial delignification and densification on chemical, morphological, and mechanical properties of wood: Structural property evolution. Ind. Crop. Prod..

[41] Prasetia D., Purusatama B.D., Kim J., Jang J., Park S., Lee S., Kim N.H. (2024). X-ray diffraction, Fourier transform infrared spectroscopy, and thermal decomposition analyses of virgin cork elements in Quercus variabilis grown in Korea. Wood Sci. Technol..

[42] Abdo M., Flity H., Terrei L., Zoulalian A., Mehaddi R., Girods P., Rogaume Y. (2024). An alternative wood pyrolysis model based on TGA and cone calorimeter tests. Thermochim. Acta.

[43] Cong R., Cai T., Ge-Zhang S., Yang H., Zhang C. (2024). Fabrication of PVA–Silica Sol Wood Composites via Delignification and Freezing Pretreatment. Polymers.

[44] Ma Y., Wei R., Zuo H., Zuo Q., Luo X., Chen Y., Wu S., Chen W. (2024). N-doped EG@MOFs derived porous carbon composite phase change materials for thermal optimization of Li-ion batteries at low temperature. Energy.

[45] Lv L., Huang S., Zhou H. (2024). Effect of introducing chemically activated biochar as support material on thermal properties of different organic phase change materials. Sol. Energy Mater. Sol. Cells.

[46] Yang K., Liu M., Du N., Huo Z., Chen Y., Yang Z., Yan P. (2024). Performance analysis of a novel phase-change wall of wood structure coupled with sky-radiation cooling. Energy Conv. Manag..

[47] Reeda V.S.J., Sakthivel S., Divya P., Javed S., Jothy V.B. (2024). Conformational stability, quantum computational (DFT), vibrational, electronic and non-covalent interactions (QTAIM, RDG and IGM) of antibacterial compound N-(1-naphthyl)ethylenediamine dihydrochloride. J. Mol. Struct..

[48] Radouane N. (2022). A comprehensive review of composite phase change materials (cPCMs) for thermal management applications, including manufacturing processes, performance, and applications. Energies.

