# Supplementary material for: The Confinement Behavior and Mechanistic Insights of Organic Phase Change Material Encapsulated in Wood Morphology Genetic Nanostructures for Thermal Energy Storage
Source: Polymers (Basel). 2024 Nov 20;16(22):3213. doi: 10.3390/polym16223213 (PMC11598059; doi:10.3390/polym16223213)
Supplement: Supplementary file 1 [file polymers-16-03213-s001.zip › polymers-3273449-supplementary.pdf]

polymers

Supplementary Materials:

The Confinement Behavior and Mechanistic Insights of Organic Phase Change Material Encapsulated in Wood Morphology Genetic Nanostructures for Thermal Energy Storage

Yang Meng *, Yanping Jiang, Yuhui Chen and Jiangyu Zhang

Yunnan Provincial Key Laboratory of Energy Saving in Phosphorus Chemical Engineering and New Phosphorus Materials, The International Joint Laboratory for Sustainable Polymers of Yunnan Province, The Higher Educational Key Laboratory for Phosphorus Chemical Engineering of Yunnan Province, Faculty of Chemical Engineering, Kunming University of Science and Technology, Kunming 650500, China; yanpingjiang@stu.kust.edu.cn (Y.J.); yuhuichen@stu.kust.edu.cn (Y.C.); zhangjiangyu@stu.edu.cn (J.Z.)
* Correspondence: mengyang@kust.edu.cn

Note S1: The Detailed Experimental Methods

1.1 Characterization Methods

1.1.1 Determination of Main Chemical Components

The main components of the modified balsa wood before and after treatment were identified using the NREL/TP-510-42618 analytical method provided by the U.S. National Renewable Energy Laboratory (NREL). The acid-insoluble lignin content was determined using a 72% sulfuric acid hydrolysis method, while the hemicellulose and α-cellulose contents were calculated based on sugar analysis using high-performance anion-exchange chromatography.

1.1.2 Microscopic Morphology

Wood powder samples were placed on conductive adhesive tape and coated with Au-Pt. The samples were then observed using a field-emission scanning electron microscope (SEM, Sigma VP, Zeiss) at an accelerating voltage of 10 kV to analyze their microscopic morphology.

1.1.3 Changes in Surface Functional Groups

Wood powder and composite phase change materials were first pressed into pellets. The samples were then placed in a Fourier-transform infrared spectrometer (FTIR, Spectrum Two, PerkinElmer) for testing, utilizing the ATR mode, with a scanning range set from 500 to 4000 cm^{-1}, 32 scans, and a resolution of 4 cm^{-1}.

1.1.4 Crystallization Behavior

Wood powder and composite phase change materials were pressed into pellets and placed on the instrument's fixed stage for detection. X-ray diffraction analysis was conducted using a Rigaku smartLab X-ray diffractometer with Cu Kα radiation (λ = 1.541 Å), at a tube voltage of 40 kV and a current of 200 mA.

1.1.5 BET Pore Size Distribution

Samples were subjected to degassing at 80°C for 2 hours in a balance chamber before being placed in the testing chamber of the instrument (Tristar II 3020, Micromeritics). Liquid nitrogen was added, and nitrogen adsorption and desorption were measured at -196°C.

1.1.6 Phase Change Performance

Samples weighing 5-10 mg were placed in a crucible under nitrogen gas protection, maintaining a flow rate of 50 mL/min. The testing was conducted using a cyclic heating-cooling mode (DSC 3+, METTLER TOLEDO). A preliminary heating-cooling cycle was completed before the normal testing to eliminate thermal errors. The temperature range was maintained from 5 to 80°C, with a heating and cooling rate of 5 °C/min. The analysis was conducted using the METTLER TOLEDO STARe Evaluation Software, which facilitates the integration of DSC data. After identifying the relevant peaks in the heat flow curves, the software automatically calculates the area under these peaks, providing both the integration limits and the corresponding thermal enthalpy values.

1.1.7 Thermogravimetric Analysis (TGA)

Thermogravimetric analysis of the modified balsa wood encapsulation carrier was performed using a TA Q500 thermogravimetric analyzer. The entire process was conducted under nitrogen protection, with a flow rate maintained at 60 mL/min. The testing temperature range was set from 30°C to 700°C, with a heating rate of 10°C/min.

1.2 Molecular Simulation

Molecular interactions between the wood-based material and phase change molecules were studied using the commercial software Material Studio 7.0. To optimize the model, cellulose was selected as the encapsulation matrix, and four molecules with the same carbon skeleton but different functional groups—n-butane, n-butanol, n-butylamine, and n-butyric acid—were chosen to simulate a series of phase change molecules with varying surface functional groups. Molecular dynamics simulations were conducted under the COMPASS force field. Initially, the geometric configuration and energy of the molecular model system were optimized to achieve equilibrium. To prevent energy accumulation, the molecular system underwent annealing in the temperature range of 200 K to 400 K. Finally, molecular dynamics simulations were performed in the canonical ensemble (NVT) for 50 ps, with a time step of 1 fs. Temperature control was implemented using the Nose-Hoover method, while the CLAYFF force field was employed for simulations, automatically assigning charges and atom types. The electrostatic interactions were calculated using the Ewald summation method, and van der Waals interactions used an atom-based summation. After the simulations, the binding energy was calculated using the formula:

$$E_{int} = E_{tot} - (E_1 + E_2), \quad (1)$$

where E_{int} is the interfacial binding energy, E_{tot} is the energy of the system composed of the carrier and phase change materials, E_1 is the energy of the isolated carrier molecule, and E_2 is the energy of the isolated phase change material.

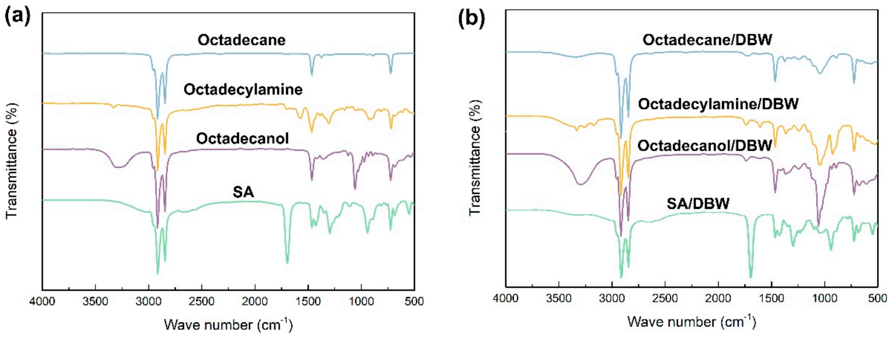

Figure S1. FTIR spectra of (a) phase change material with different functional groups and (b) the as-prepared composite.

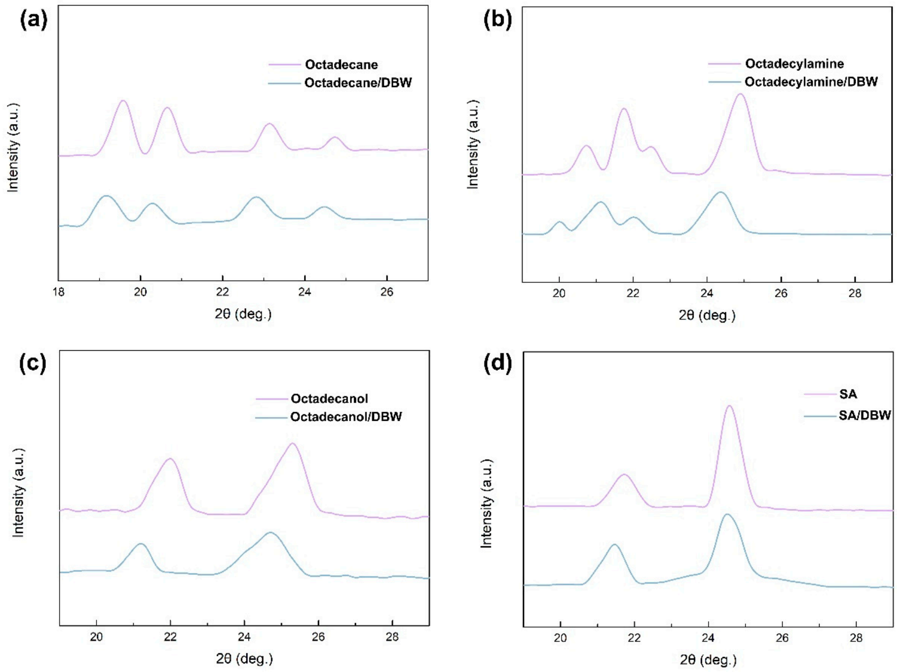

Figure S2. XRD curves of fatty acid and as-prepared composite phase materials: (a) Octadecane, (b) Octadecylamine, (c) Octadecanol, and (d) Stearic acid (SA).

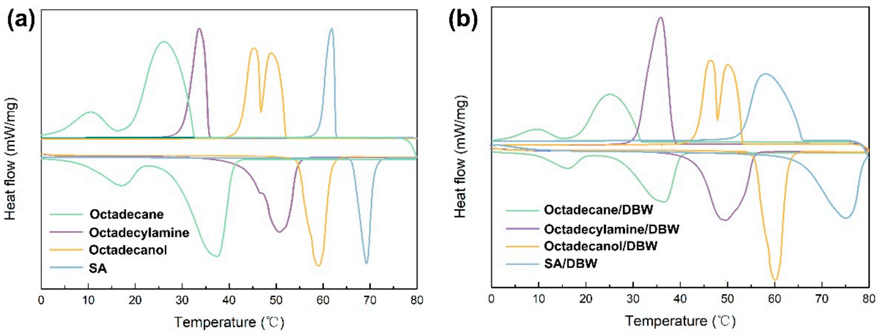

Figure S3. DSC of (a) phase change material with different functional groups and (b) the as-prepared composite.

Table S1. The thermal physical properties of pure organic phase change materials

PCMs	$\triangle H_m$ (J/g)	$\triangle H_f$ (J/g)
LA	160.26±0.51	167.53±0.50
MA	172.44±0.47	175.97±0.49
PA	183.85±0.56	181.81±0.61
SA	202.45±0.73	204.78±0.68
Octadecanol	226.26±0.67	228.81±0.63
Octadecylamine	224.36±0.58	243.57±0.57
Tetradecane	200.11±0.77	202.34±0.74
Hexadecane	210.67±0.57	212.65±0.56
Octadecane	220.88±0.71	222.22±0.77

Note: $\triangle H_m$ represents the melting enthalpy; $\triangle H_f$ represents the freezing enthalpy.

Table S2. The thermal physical properties of wood-based CPCMs

Wood-based CPCMs	$\triangle H_m$ (J/g)[1]	$\triangle H_f$ (J/g)[1]	Crystallinity (F_c, %)
LA/DBW	62.11±0.27	61.76±0.21	65.14
MA/DBW	76.36±0.22	75.73±0.20	76.67
PA/DBW	88.46±0.33	87.12±0.29	78.44
SA/DBW	81.11±0.25	80.09±0.22	66.65
Octadecanol/DBW	91.54±0.35	90.77±0.32	67.77
Octadecylamine/DBW	92.22±0.32	91.07±0.31	69.65
Tetradecane/DBW	103.77±0.35	102.01±0.32	85.58
Hexadecane/DBW	112.20±0.43	111.12±0.39	89.98
Octadecane/DBW	98.09±0.21	97.88±0.20	73.77

[1] Note: $\triangle H_m$ and $\triangle H_f$ represent the melting and crystallization enthalpies of both PCM and CPCMs, respectively.

Table S3. PCMs molecular sizes [1]

PCM Molecule	Chemical Formula	Carbon Chain Length	Average Molecular Size (nm)
Lauric Acid (LA)	$C_{12}H_{24}O_2$	12	1.6
Myristic Acid (MA)	$C_{14}H_{28}O_2$	14	1.8
Palmitic Acid (PA)	$C_{16}H_{32}O_2$	16	2.0
Stearic Acid (SA)	$C_{18}H_{36}O_2$	18	2.2
Octadecanol	$C_{18}H_{38}O$	18	2.2
Octadecylamine	$C_{18}H_{39}N$	18	2.2
Tetradecane	$C_{14}H_{30}$	14	1.7
Hexadecane	$C_{16}H_{34}$	16	1.9
Octadecane	$C_{18}H_{38}$	18	2.1

Table S4. The bonding energy of PCMs and cellulose

Bimolecular Combinations	E_1 (kcal/mol)	E_2 (kcal/mol)	E_{tot} (kcal/mol)	E_{int} (kJ/mol)
Butane/cellulose	-6.977	-1175.819	-63.698	2302
Butylamine/cellulose	-25.729	-1175.819	-194.121	2209
Butanol/cellulose	-18.744	-1175.819	-345.856	2040
Butyric Acid/cellulose	-20.542	-1175.819	-543.753	1847

References

1. Radouane, N. A comprehensive review of composite phase change materials (cPCMs) for thermal management applications, including manufacturing processes, performance, and applications. Energies **2022**, *15*(21), 8271.
